# A complete reference genome of broomcorn millet

**DOI:** 10.1038/s41597-024-03489-5

**Published:** 2024-06-21

**Authors:** Haigang Wang, Junjie Wang, Chunhai Chen, Ling Chen, Meng Li, Huibin Qin, Xiang Tian, Sen Hou, Xukui Yang, Jianbo Jian, Peng Gao, Lun Wang, Zhijun Qiao, Zhixin Mu

**Affiliations:** 1 Center for Agricultural Genetic Resources Research, Shanxi Agricultural University, Key Laboratory of Crop Gene Resources and Germplasm Enhancement on Loess Plateau, Ministry of Agriculture and Rural Affairs, Taiyuan, 030031 China; 2 BGI Genomics, Shenzhen, 518083 China; 3 BGI, Shenzhen, 518083 China

**Keywords:** Plant molecular biology, Agricultural genetics

## Abstract

Broomcorn millet (*Panicum miliaceum* L.), known for its traits of drought resistance, adaptability to poor soil, short growth period, and high photosynthetic efficiency as a C_4_ plant, represents one of the earliest domesticated crops globally. This study reports the telomere-to-telomere (T2T) gap-free reference genome for broomcorn millet (AJ8) using PacBio high-fidelity (HiFi) long reads, Oxford Nanopore long-read technologies and high-throughput chromosome conformation capture (Hi-C) sequencing data. The size of AJ8 genome was approximately 834.7 Mb, anchored onto 18 pseudo-chromosomes. Notably, 18 centromeres and 36 telomeres were obtained. The assembled genome showed high quality in terms of completeness (BUSCO score: 99.6%, QV: 61.7, LAI value: 20.4). In addition, 63,678 protein-coding genes and 433.8 Mb (~52.0%) repetitive sequences were identified. The complete reference genome for broomcorn millet provides a valuable resource for genetic studies and breeding of this important cereal crop.

## Background & Summary

Broomcorn millet (*Panicum miliaceum* L.), a member of the Paniceae tribe in the Gramineae family, exhibits remarkable adaptability to marginal regions due to its short growing season (60–90 days), low water requirements, high salt tolerance, and efficient nutrient resource utilization^1,2^. Being a C_4_ plant, broomcorn millet demonstrates enhanced carbon fixation and efficient utilization of water and nitrogen resources. Additionally, its grains are characterized by their gluten-free nature and exceptional nutritional value, containing higher protein content, mineral composition, and antioxidant levels compared to most other cereals^3^. Consequently, broomcorn millet has been extensively cultivated in semiarid regions across Asia, Europe, and other continents and is considered one of the oldest crops worldwide^4^. The cultivation of broomcorn millet holds promise for enhancing food security, diversifying agriculture, and promoting a healthier diet^5^. Broomcorn millet has an allotetraploid genome consisting of 36 (2n = 4x = 36) chromosomes^6^. Although four chromosome-level of broomcorn millet, Jinshu7^7^, LM_v1^8^, LM_v2^9^, and Pm_0390^8^, have been made available, there are still missing segments within the genome due to the presence of highly repetitive sequences clustered across the genome, particularly in the telomere and centromere regions. In recent years, T2T and gap-free genomes have been successfully obtained in various important crops, including rice^10^, barley^11^, and maize^12^.

**Fig. 1:**
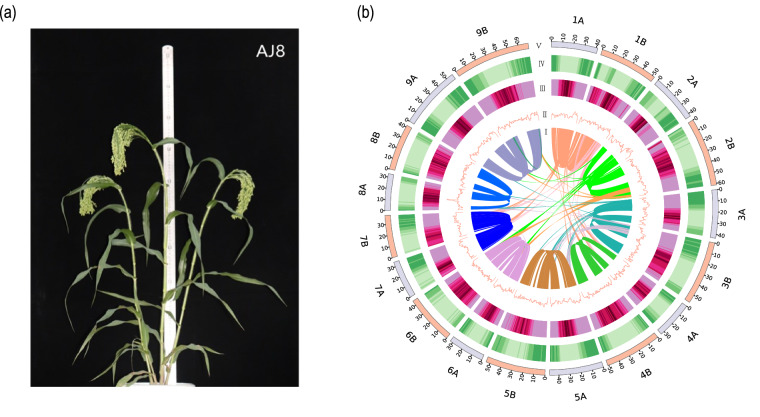
An overview of the AJ8 genome. (**a)** The photograph of the AJ8 plant. **(b)** Circos plot illustrating the genome of the AJ8 genome. The plot includes the following components, arranged from inside to outside: (I) Collinear regions within the AJ8 assembly; (II) GC content in non-overlapping 1 Mb windows; (III) Percentage of repeats in 1-Mb sliding windows; (IV) Gene density in 1-Mb sliding windows; (V) Length of pseudo-chromosome in megabases (Mb).

In the present study, we assembled the first T2T gap-free genome of broomcorn millet (AJ8) (Fig. 1a), achieved through PacBio HiFi long reads, Nanopore technologies and Hi-C sequencing data. The resulting complete genome assembly has a final size of 834.7 Mb and is organized into 18 pseudo-chromosomes (Table 1; Fig. 1b). Gene annotation identified 52.0% repetitive sequences and 63,678 protein-coding genes (Fig. 1b). This complete reference genome provides a robust foundation for future studies on population and conservation genetics of broomcorn millet.

**Table 1:** Summary statistics of broomcorn millet genome assemblies.

Item	This study (AJ8)	Wang *et al*.^9^ (LM_v2)	Sun *et al*.^7^ (Jinshu7)	Zou *et al*.^57^ (Pm_0390)	Shi *et al*.^8^ (LM_v1)
Size of assembly (Mb)	834.7	846.0	862.0	854.8	848.4
Number of contigs	18	704	4,062	5,538	1,308
Number of scaffolds	18	444	1,616	1,306	475
Contig N50 (Mb)	48.3	26.2	0.7	0.4	2.6
GC content	46.8%	46.8%	45.7%	45.9%	46.3%
Anchored pseudo-chromosomes (%)	100.0	99.0	93.3	96.2	98.9
Number of gaps in chromosomes	0	304	2,447	4,232	829
LTR assembly Index	20.4	20.8	11.1	9.3	15.9
Genome complete BUSCOs	99.6%	98.9%	98.7%	99.0%	98.8%
Quality value	61.7	—	—	—	—
Repetitive sequences	52.0%	57.9%	57.6%	58.2%	54.1%
Number of protein-coding genes	63,678	60,096	61,782	55,930	63,671

## Methods

### Plant materials and growth conditions

The broomcorn millet landrace sequenced in this study was originally collected from Center for Agricultural Genetic Resources Research, Shanxi Agricultural University, Taiyuan, Shanxi Province (coordinates: E 112° 34′ 26.66″, N 37° 46′ 37.16″). The plants were planted under controlled conditions with a temperature of 25 °C, humidity of 60%, and a light intensity following a 14-hour day and 10-hour night cycle. Twenty seedlings with consistent growth at the fourth leaf stage were carefully chosen and sampled from various organs, including roots, stems, and leaves. A weight of 2 g was measured for each tissue organ, which was immediately placed in a freezing chamber with liquid nitrogen and subsequently stored at −80 °C.

### Long insert libraries preparation and sequencing

Genomic DNA was extracted from leaf tissue using DNeasy Plant Maxi kit (Qiagen). The PacBio long insert libraries were prepared according to manufacturers’ instructions with an insert size of approximately 20 kb (Pacific Biosciences, USA). Subsequently, the libraries were subjected to sequencing using PacBio Sequel II platforms in circular consensus sequencing mode. The subreads were processed using SMRTLink (v11.1.0)^13^ with parameters “–minPasses 3 –minPredictedAccuracy 0.99 –minLength 500”, yielding approximately 77.0 Gb high-fidelity (HiFi) reads with a N50 size of about 18.0 kb (Table 2).

**Table 2:** Summary of sequencing data of AJ8 genome.

	Reads Number	Total length (bp)	Genome depth	N50 length of reads (bp)
Total ultra-long ONT data	5,657,711	168,590,000,000	196	55,765
Total error corrected ONT data	364,289	34,765,836,136	40	92,975
PacBio subreads	69,659,269	1,153,616,500,153	1338.3	18,495
PacBio CCS reads	4,321,122	77,005,593,171	89.3	17,995
Hi-C raw data	1,700,452,074	255,067,811,100	295.9	150
Hi-C clean data	1,686,533,526	252,980,028,900	293.5	150

The ONT ultra-long insert libraries were generated using the Oxford Nanopore SQK-LSK109 kit, and then sequenced on a PromethION flow cell (Oxford Nanopore Technologies, Oxford, UK). A total of 165.6 Gb of ONT data with 196x coverage was generated, and the N50 value was 55,765 bp (Table 2). After error correction and length filtering of the data, 34.8 Gb ultra long ONT reads with the N50 value 92, 975 bp were obtained (Table 2).

### Short insert libraries preparation and sequencing

For chromosomal conformational capture (Hi-C) sequencing, Hi-C libraries based on *Dpn*II restriction enzymes were generated as previously described^14^, and sequenced on the MGISEQ-2000 platform. A total of 253.0 Gb of clean data were obtained from 255.1 Gb of sequencing data using software SOAPnuke (v2.0)^15^ with parameters “-n 0.01 -l 20 -q 0.1 -i -Q 2 -G 2 -M 2 -A 0.5” (Table 2).

RNA-seq libraries from leaf tissues were constructed using the NEBNext® Ultra™ RNA Library Prep Kit for Illumina® (NEB, Ipswich, MA, USA) following the manufacturer’s protocol. Then the RNA libraries were sequenced on a MGISEQ-2000 instrument and generated 150 bp paired-end reads. After quality control by fastp (0.19.5)^16^ with parameters of “–adapter_sequence AAGTCGGAGGCCAAGCGGTCTTAGGAAGACAA–adapter_sequence_r2 AAGTCGGATCGTAGCCATGTCGTTCTGTGAGCCAAGGAGTTG–average_qual 15 -l 150”, each library contained more than 7.8 Gb of clean data. More than 98.1% of the clean data had scores greater than Q20 in each library (Table 3).

**Table 3:** Summary of RNAseq sequencing data of AJ8 genome.

Sample	Develoamental stage	Tissue	Total raw reads (Mb)	Total clean reads (Mb)	Total clean bases (Gb)	Clean reads Q20%	Clean reads GC%	Total MappingRatio
SL	seedling	leaf	60.30	57.99	8.70	98.29	57.23	99.33%
SS	seedling	stem	67.87	64.24	9.64	98.48	55.79	99.28%
SR	seedling	root	67.81	64.69	9.70	98.62	55.30	98.98%
TL	tillering	leaf	63.40	60.46	9.07	98.40	56.82	99.21%
TS	tillering	stem	69.89	66.85	10.03	98.53	55.04	99.26%
TR	tillering	root	72.38	67.93	10.19	98.71	54.32	92.81%
JL	jointing	leaf	56.91	54.30	8.14	98.45	56.73	99.33%
JS	jointing	stem	72.59	68.77	10.31	98.54	55.06	99.28%
JR	jointing	root	54.08	52.05	7.81	98.44	54.60	97.20%
BL	booting	leaf	72.88	69.47	10.42	98.43	57.06	99.24%
BS	booting	stem	58.16	55.33	8.30	98.42	55.94	99.28%
BR	booting	root	62.12	60.13	9.02	98.60	55.20	98.32%
BT	booting	tassel	61.17	58.46	8.77	98.53	55.11	99.21%
HL	heading	leaf	64.09	61.15	9.17	98.49	56.79	99.26%
HS	heading	stem	65.65	61.42	9.21	98.44	56.33	99.37%
HR	heading	root	64.52	61.84	9.28	98.59	54.38	97.12%
HT	heading	tassel	68.19	64.74	9.71	98.36	55.19	99.19%
FL	fillling	leaf	62.43	59.36	8.90	98.46	55.76	99.34%
FS	fillling	stem	60.22	58.35	8.75	98.48	54.05	99.07%
FR	fillling	root	75.01	72.73	10.91	98.44	54.34	98.88%
FT	fillling	tassel	57.27	54.80	8.22	98.12	53.80	99.19%

### Genome assembly

Using HiFi reads, ultra-long ONT reads, and Hi-C clean data, the primary contigs were assembled by Hifiasm (v 0.19.5)^17^ with default parameters. To anchor contigs onto chromosomes, we used BWA (v 0.7.12)^18^ to align the Hi-C clean data to the assembled contigs. Low-quality reads were filtered out using the HiC-Pro pipeline^19^ with default parameters. The remaining valid reads were employed to anchor chromosomes with Juicer^20^ and 3d-dna pipeline^21^. Excitingly, our results showed that the hifiasm assembly consists of contiguous sequences covering the entire length of all chromosomes. This achievement can be attributed to the remarkable accuracy of HiFi data, the utilization of ultra-long ONT data, and the ongoing enhancements in assembly algorithms. Analogous to the T2T genome of rapeseed^22^, the hifiasm assembly comprises continuous sequences spanning the entirety of nine chromosomes. For further refinement of the genome, the T2T assembly was polished using a similar method described by Mc Cartney *et al*.^23^. In brief, the HiFi reads were aligned to the T2T assembly using Winnowmap2 (v 2.03)^24^. The resulting alignments were filtered to exclude secondary alignments and alignments with excessive clipping by using ‘falconc bam-filter-clipped’ tool. Finally, racon (v 1.5.0)^25^ was performed with the filtered alignments. The final assembled genome had a length of 834,678,208 bp and a contig N50 of 48.3 Mb (Table 1). The assembled sequences were successfully anchored to 18 pseudo-chromosomes (Table 1). The completeness of the assembly was assessed using Benchmarking Universal Single-Copy Orthologs (BUSCO) (v 5.5.0)^26^ with the embryophyta_odb10 (parameters: -m genome -l embryophyta_odb10). We appylied Merqury (v1.3)^27^ using PacBio HiFi long reads with a *K*-mer value of 17-bp to estimate the quality value.

### Annotation of repetitive sequences

Tandem repeats and interspersed repeats were identified using the method described in Qu *et al*.^28^. In brief, RepeatModeler (v1.0.4)^29^ and LTR-FINDER (v1.0.7)^30^ were employed to construct of a *de novo* repeat sequence library. This compiled library was subsequently deployed for the detection of interspersed repeats and low-complexity sequences utilizing RepeatMasker (v4.0.7)^31^. DNA and protein transposable elements (TEs) were identified through RepeatMasker (v4.0.7) and RepeatProteinMasker (v4.0.7), respectively. Tandem repeats were discerned utilizing Tandem Repeat Finder (v4.10.0)^32^. A total of 433.8 Mb (~52.0%) of repetitive sequences were obtained. Among the interspersed repeats, three types of repetitive elements, namely class I (retrotransposons), class II (DNA transposons), and unclassified elements, accounted for 49.6% of the genome assembly (Table 4). The telomeric sequences and centromere region in the AJ8 genome assembly were identified using quartet (v1.1.36)^33^ with “-c plant”.

**Table 4:** Interspersed repeat contents in AJ8 genome assembly.

Type	Repbase TEs		TE protiens		*De novo*		*Combined TEs	
	Length (bp)	% in genome	Length (bp)	% in genome	Length (bp)	% in genome	Length (bp)	% in genome
**Class I**	209,289,045	25.07	108,867,424	13.04	347,247,904	41.60	379,892,897	45.51
LTR	199,720,006	23.93	102,637,122	12.30	341,764,732	40.95	367,377,405	44.01
Gypsy	167,578,773	20.08	83,383,157	9.99	303,670,978	36.38	321,394,835	38.51
Copia	31,228,868	3.74	19,222,359	2.30	32,348,058	3.88	44,339,339	5.31
LINE	9,720,644	1.16	6,231,875	0.75	5,688,415	0.68	13,406,696	1.61
SINE	151,212	0.02	0	0.00	39,826	0.00	186,663	0.02
**Class II: DNA**	30,371,375	3.64	6,938,097	0.83	13,865,556	1.66	38,069,361	4.56
**†Unclassified**	11,035	0.00	0	0.00	2,576,970	0.31	2,588,002	0.31
**Total**	239,065,404	28.64	115,803,473	13.87	362,426,030	43.42	414,237,138	49.63

Note: This statistical table does not contain Tandem Repeats, some elements may partly include another element domain.

^*^Combined: the non-redundant consensus of all repeat prediction/classification methods employed.

^†^Unclassified: the predicted repeats that cannot be classified by RepeatMasker.

LINE, long interspersed nuclear elements; SINE, short interspersed nuclear elements; LTR, long terminal repeat.

### Protein-coding genes prediction and functional annotation

Gene prediction in this study was conducted using a combination of transcriptome-based prediction, homology-based prediction, and *ab initio* prediction methods. For transcriptome-based prediction, 194.2 Gb Illumina clean reads from 21 samples were assembled by Trinity (v 2.15.1)^34^ with parameters of ‘–max_memory 200 G–CPU 40–min_contig_length 200–genome_guided_bam merged_sorted.bam–full_cleanup–min_kmer_cov 4–min_glue 4–bfly_opts ‘-V 5–edge-thr = 0.1–stderr’–genome_guided_max_intron 10000’, which generated 227,501 transcripts with a N50 of 2,698. These assembled transcripts were aligned against the AJ8 T2T assembly using Program to Assemble Spliced Alignment (PASA) (v 2.4.1)^35^. Gene structures were generated from valid transcript alignments (PASA-set). RNA-seq clean reads were also mapped to the AJ8 T2T assembly using Hisat2 (v 2.0.1)^36^. Stringtie (v 1.2.2)^37^ and TransDecoder (v 5.7.1) (https://github.com/TransDecoder/TransDecoder) were employed to assemble the transcripts and identify candidate coding regions into gene models (Stringtie-set). Homologous genomes from seven plants, including rice (T2T-NIP)^10^, maize (T2T Mo17)^12^, *A. thaliana* (Col-PEK)^38^, sorghum (GCA_000003195.3)^39^, pearl millet (Tift23D2B1-P1-P5)^40^, foxtail millet (GCA_000263155.2)^41^, and two previous versions of broomcorn millet (Jinshu7: GCA_026771285.1; Longmi4: GCA_002895445.3)^7,9^ were downloaded and used as queries to search against the AJ8 T2T assembly using GeMoMa (v 1.9)^42^. These homology predictions were referred to as “Homology-set”. For *ab initio* prediction methods, AUGUSTUS (v 3.2.3)^43^ was used to predict coding regions in the repeat-masked genome. All gene models were combined using EvidenceModeler (v 2.1.0)^44^ with different weight parameters assigned to evidence from different sources (10 for PASA-set, 5 for Stringtie-set, 5 for Homology-set, and 1 for AUGUSTUS gene prediction). The resulting protein-coding genes that were only derived from *ab initio* prediction were filtered out. Finally, the produced gene models were further refined with PASA (v 2.4.1)^37^ to generate untranslated regions and alternative splicing variation information. The final comprehensive gene set comprised 63,678 genes.

The integrated gene set was translated into amino-acid sequences and annotated using the method described in Zhou *et al*.^45^. As a result, 98.8% of the predicted protein-coding genes were functionally annotated (Table 5). In addition, we utilized the Diamond (v 0.9.30)^46^ software (E value <= 1e-05) to perform homologous comparison analyses between AJ8 and *Arabidopsis* (TAIR10)^47^, as well as between AJ8 and rice (Osativa_v7_0)^48^. Among the AJ8 genes, 48,284 (75.83%) genes exhibited homology with *Arabidopsis*, while 56,777 (89.16%) genes showed homology with rice (Table S1).

**Table 5:** Number of functional annotations for predicted genes in AJ8 assembly.

Type		Gene number	Percentage
Total		63,678	100.00%
NR		61,980	97.33%
SwissProt		44,342	69.63%
KEGG		43,826	68.82%
KOG		42,801	67.21%
TrEMBL		61,821	97.08%
Interpro	All	60,143	94.45%
	GO	35,547	55.82%
Annotated		62,885	98.75%
Unannotated		793	1.25%

### Gene expression analysis

The gene expression analysis was used the same method as previously reported^49^. The expression heatmap was constructed using heatmap R package. The expression matrix of genes in different transcriptome samples was displayed in Table S2.

### Synteny analysis

The identification of syntenic regions was based on conducting homology searches using MCScan (Python version)^50^, with a minimum requirement of 30 genes per block.

### Subgenome phasing

By employing repetitive k-mers as “differential signatures” and utilizing the SubPhaser software, we successfully phased the subgenomes of AJ8. The results obtained from SubPhaser^51^ were found to be consistent with the Jinshu7 genome, as indicated in Table 6 and Fig. 4.

**Table 6:** Correspondence between chromosome identificationss of AJ8 and Jinshu7.

Primary ID	New ID after subgenome phaser
Chr01	9B
Chr02	2B
Chr03	3B
Chr04	9 A
Chr05	5B
Chr06	1B
Chr07	4B
Chr08	5 A
Chr09	6B
Chr10	3 A
Chr11	2 A
Chr12	1 A
Chr13	8B
Chr14	7B
Chr15	7 A
Chr16	4 A
Chr17	8 A
Chr18	6 A

^#^Consistency of AJ8 new ID with Jinshu7 Genome.

## Data Records

The sequencing data and assembled genome sequence have been deposited in the Sequence Read Archive with accession numbers SRP482566^52^ under project number PRJNA1059665. The genome assembly has been deposited at GenBank under the WGS accession

GCA_038442765.1^53^. Files of the gene structure annotation, repeat predictions and gene functional annotation were deposited at Figshare^54^.

## Technical Validation

### Genome assembly and gene prediction quality assessment

**Fig. 2:**
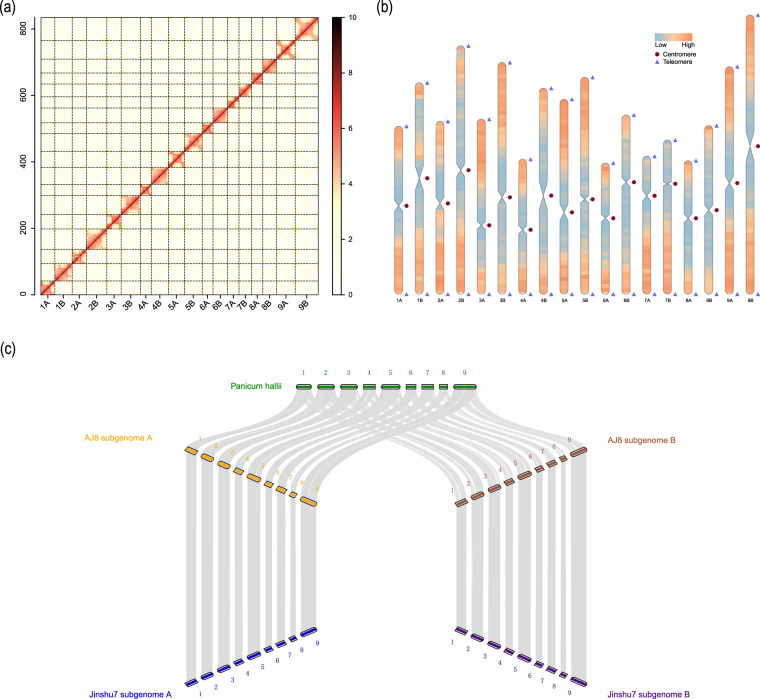
The high-quality of the AJ8 genome. (**a)** Heatmap displaying Hi-C interactions of AJ8 pseudomolecules. **(b)** Telomere detection map. Triangles and circles represent telomeres and centromere within the AJ8 assembled chromosomes. The orange color represents regions with high gene density, while the dark sky blue color represents regions with low gene density. **(c)** Synteny analysis of *Panicum hallii*, AJ8 and Jinshu7.

The accuracy and integrity of AJ8 T2T assembly were assessed through several analyses. Firstly, the Hi-C heatmap displayed consistent results across all chromosomes, indicating the correct ordering and orientation of contigs in the assembly (Fig. 2a). Secondly, the assembly successfully captured 18 centromeres and all 36 telomeres, providing strong evidence for their integrity (Fig. 2b; Tables 7, 8). Thirdly, the assembly showed high collinearity with Jinshu7 (GCA_026771285.1)^7^ and *Panicum hallii* (GCA_002211085.2)^55^ (Fig. 2c). Fourthly, alignment results from minimap2 (v 2.24-r1122)^56^ revealed that 100.0% of ONT reads and 99.98% of HiFi reads could be aligned to the AJ8 T2T assembly. Additionally, the average genome alignment rate of the transcriptome was 98.7% (Table 3). Lastly, AJ8 T2T demonstrated an LTR assembly index of 20.4, a quality value of 61.7, and a BUSCO score of 99.6%, indicating its high completeness (Table 1).

**Table 7:** The identified telomeres in AJ8 assembly.

Chromosomes	Length (bp)	Ends identified with telomeres	Total counts of telomere repeats at the left end	Total counts of telomere repeats at the right end
1 A	41,558,686	both	2,002	2,776
1B	52,342,684	both	2,317	2,037
2 A	42,791,896	both	578	504
2B	61,479,593	both	2,843	767
3 A	43,337,656	both	13	1,655
3B	57,374,001	both	2,860	2,883
4 A	33,404,080	both	1,063	1,428
4B	50,983,745	both	1,828	2,685
5 A	48,261,264	both	14	2,623
5B	53,707,716	both	3,011	16
6 A	32,475,619	both	2,582	2,535
6B	44,372,877	both	179	3,933
7 A	34,166,222	both	12	2,776
7B	38,174,571	both	13	788
8 A	33,047,006	both	1,857	3,687
8B	41,728,197	both	2,085	2,425
9 A	56,346,356	both	1,265	1,706
9B	69,126,039	both	1,563	1,747

**Table 8:** The distribution of centromeres in AJ8 assembly.

Chromosomes	Length (bp)	Start	End	Centro Length (bp)	TR percentage
1 A	18,342,834	21,040,163	2,697,330	1,583,268	58.70%
1B	20,653,421	26,624,068	5,970,648	1,298,067	21.74%
2 A	18,772,875	21,884,089	3,111,215	836,409	26.88%
2B	29,257,159	32,333,321	3,076,163	808,436	26.28%
3 A	25,401,874	27,185,391	1,783,518	878,557	49.26%
3B	31,759,679	35,045,521	3,285,843	985,466	29.99%
4 A	16,454,400	18,471,716	2,017,317	1,152,860	57.15%
4B	23,164,795	29,986,713	6,821,919	2,358,195	34.57%
5 A	25,993,962	30,048,990	4,055,029	1,449,363	35.74%
5B	29,311,126	31,111,890	1,800,765	378,448	21.02%
6 A	12,733,711	14,619,504	1,885,794	1,351,381	71.66%
6B	15,559,124	17,754,000	2,194,877	775,006	35.31%
7 A	8,527,110	11,079,922	2,552,813	969,677	37.98%
7B	10,357,744	11,349,473	991,730	190,239	19.18%
8 A	13,176,836	15,335,732	2,158,897	1,114,728	51.63%
8B	19,654,938	22,202,119	2,547,182	484,580	19.02%
9 A	27,161,481	30,534,321	3,372,841	1,953,960	57.93%
9B	28,766,226	36,231,841	7,465,616	910,724	12.20%

**Fig. 3:**
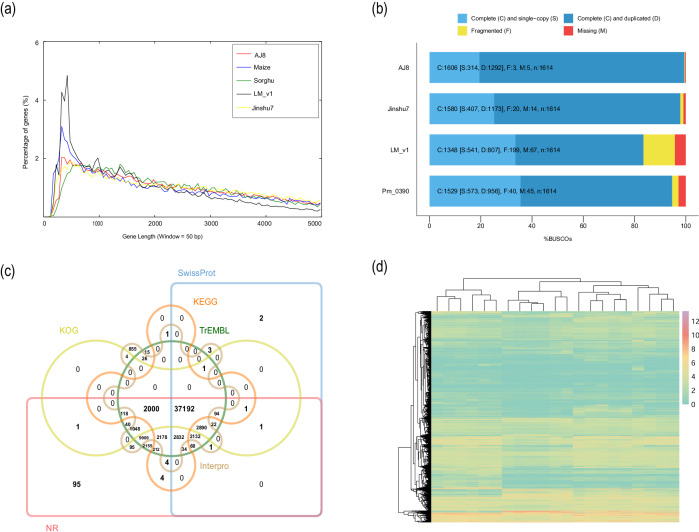
Gene prediction quality assessment. (**a)** The composition of gene length in the AJ8 genome compared to the genomes of other species. **(b)** BUSCO assessments of the AJ8 gene. **(c)** Venn diagram showing the number of genes with homology or functional classification by each method. **(d)** The expression heatmap illustrates the expression levels among 21 RNA-seq samples. The color bar in the lower right corner represents log2-transformed FPKM values. Blue and red boxes indicate genes with lower and higher expression levels, respectively.

We compared the length distribution of genes among the AJ8, maize^12^, sorghum (GCF_000003195.3)^39^, LM_1^8^, and Jinshu7^7^ and found similar patterns (Fig. 3a). The BUSCO analysis showed that 99.5% (single-copy gene: 19.5%, duplicated gene: 80.0%) of 1,614 embryophyta single-copy orthologs were successfully identified as complete, while 0.2% were fragmented and 0.3% were missing in the assembly (Fig. 3b). 62,885 (98.8%) gene models were successfully annotated in diverse databases and 51,958 gene models (81.6%) exhibited detectable transcriptional activity (FPKM value ≥ 1) across 21 RNA-seq samples (Fig. 3c; Table 5; Fig. 3d). Taken together, these results provide strong evidence that a high-quality AJ8 genome has been obtained. The high-quality genome provides a solid foundation for uncovering the drought resistance and adaptive mechanisms of AJ8, and also serves as an important reference for the rapid breeding of AJ8 and other crops.

**Fig. 4:**
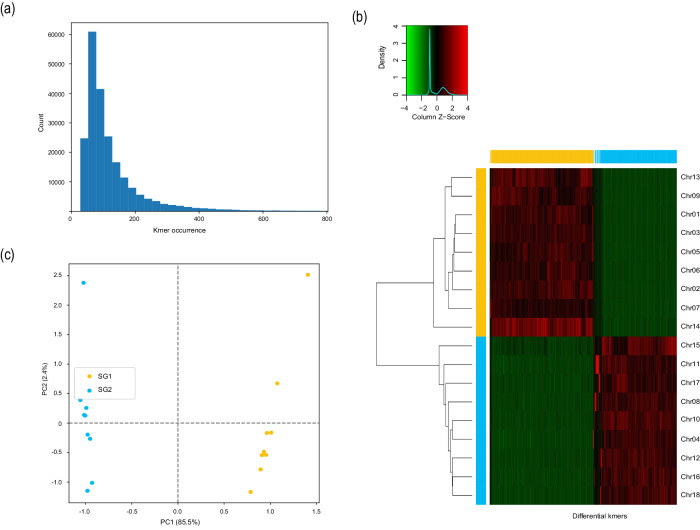
Phased subgenomes of the AJ8 genome. (**a)** The histogram of differential k-mers among homoeologous chromosome sets. **(b)** Heatmap and clustering of differential k-mers. The x-axis, differential k-mers; y-axis, chromosomes. The vertical color bar, each chromosome is assigned to which subgenome; the horizontal color bar, each k-mer is specific to which subgenome (blank for non-specific kmers). **(c)** Principal component analysis of differential k-mers. Points indicate chromosomes.

### Supplementary information

Table S1

Table S2

## Data Availability

No specific code or script was used in this work. Commands used for data processing were all executed according to the manuals and protocols of the corresponding software.
